# The production of ignorance about medication abortion in Tunisia: between state policies, medical opposition, patriarchal logics and Islamic revival

**DOI:** 10.1016/j.rbms.2021.11.001

**Published:** 2021-11-15

**Authors:** Irene Maffi

**Affiliations:** STSlab, Institute of Social Sciences, Faculty of Social and Political Sciences, University of Lausanne, Lausanne, Switzerland

**Keywords:** Healthcare providers, Ignorance, Law, Medication abortion, Opposition, Tunisia

## Abstract

In Tunisia, medication abortion has been available in government reproductive and sexual health clinics since the early 2000s. Since its introduction, it has rapidly replaced the surgical method, and between 75% and 80% of abortions in the public sector were performed using the pharmacological protocol in 2016. In this article, I intend to discuss the various forms of ignorance about medication abortion that exist in Tunisia among several categories of actors in relation to the legal, medical and religious domains. I explore how the existing ‘varieties of ignorance’ are related to the specific political, social and economic positions of the involved actors, the dominant gender regime, specific institutional policies and economic interests. I also investigate how some forms of ignorance are wilfully produced by institutions and individuals, whereas others are the result of positionality or organizational features. I first describe when and how medication abortion was introduced in Tunisia and the forms of resistance it elicited; later, I examine the production of ignorance about this technology after the revolution of 2011. I mainly consider practices and discourses of health professionals, but also those of women seeking abortion care in the public sector, and those of the activists of a Tunisian non-governmental organization operating in the domain of women’s health and rights.

## Introduction

In Tunisia, medication abortion has been available in government reproductive and sexual health clinics since the early 2000s. After its introduction, it rapidly replaced the surgical method; in 2016, between 75% and 80% of abortions in the public sector were performed using the pharmacological protocol (Avortement médicamenteux: 15 ans d’innovations au service de la femme en Tunisie, 2016). According to the only available report, abortions in the private sector are between one and a half to three times more numerous than in public facilities, and most abortions are performed using surgical methods (Ben Hamida, 2011). The number of abortions in the public sector has remained stable since the introduction of the pharmacological method (Avortement médicamenteux: 15 ans d’innovations au service de la femme en Tunisie, 2016), but seems to have increased in private clinics over the 2000s due to the suppression of this service in many government facilities (Association tunisienne des femmes démocrates, 2013). Although healthcare providers of public and private facilities offering abortion care received state-sponsored training on medication abortion protocol in the years since the revolution of 2011, abortion practices and information were often incorrect. This was also an effect of the overthrow of Ben Ali’s rule. The revolution – which originated as an economic and political protest against the authoritarian regime of Ben Ali – allowed the legalization of the once-banned Islamist party Ennahdha that won the first democratic elections in October 2011. The party remained in power until early 2014 and had a majority in the Constituent Assembly where the new constitution was being discussed. During the first years of the revolution, very conservative views of Islam spread freely in the country, and several civil rights were questioned, especially women’s rights. Equality between men and women was put into question, and in January 2013, Najiba Berioul, a deputy of Ennahdha, explicitly contested the right to abortion, although the law was not changed. In addition, state control over public institutions was weak and many health professionals working in government facilities began to behave according to their personal beliefs rather than the rules of the Ministry of Health; for example, turning away women coming for abortion care (Hajri et al., 2015, Maffi, 2020).

Upon the introduction of medication abortion in the country, despite clinical studies proving its safety, many Tunisian doctors opposed its use, allegedly because they considered it to be dangerous to women’s health. Some practitioners expressed concerns about the possible banalization of the act and its moral and social consequences, and others opposed the use of pharmacological treatment because it makes women less dependent on experts’ knowledge. Several health providers I met misinformed women seeking abortion care about the action and effects of medication abortion, either intentionally or unintentionally, and international pharmacological protocols are often not respected in the private sector. The apparent paradox of the production of ignorance about medication abortion, a widespread, legal practice in Tunisia, is rooted in several diverse logics. As I will show, it is mainly the result of pervasive ideological opposition to (medication) abortion, which emerged in the 2000s in relation to changes in government family planning policies, severe cuts in the public health system (Foster, 2016, Maffi, 2020), and the increased religious conservatism that culminated in the suppression of abortion services in many facilities after the revolution.

In this article, I intend to discuss the various forms of ignorance about medication abortion in Tunisia among several categories of actors in relation to the legal, medical and religious domains. I will explore how the existing ‘varieties of ignorance’ (Abbott, 2010) are linked to the specific political, social and economic positions of the involved actors, the dominant gender regime, specific institutional policies and economic interests. I intend to investigate how some forms of ignorance are wilfully produced by institutions and individuals, whereas others are the result of positionality and systemic or organizational features (Dedieu and Jouzel, 2015, McGoey, 2012). Ignorance in the latter sense is not a deviation but a ‘regular’ (Gross and McGoey, 2015) aspect of human organizations and societies. I will first describe when and how medication abortion was introduced in Tunisia and the forms of resistance it elicited; later, drawing on my ethnographic material, I will examine the production of ignorance about this technology since the revolution of 2011. I will mainly consider practices and discourses of health professionals, but also those of women seeking abortion care in the public sector, and those of the activists of a Tunisian non-governmental organization (NGO) operating in the domain of women’s health and rights. Public institutions in charge of sexual and reproductive care and private clinics offering abortion care will be taken into consideration. Finally, I will show how new conservative Islamic positions about abortion have also played a role in producing ignorance about this technology among birth practitioners and patients alike.

## Materials and methods

Based on 10 months of fieldwork in Tunisia (August 2013–June 2014), the research took place in the area of Great Tunis which includes four governorates (out of 24) and covers a mainly urban area. I completed participant observation in three government reproductive and sexual health clinics, and in the family planning unit of a large public hospital. These medical facilities were among the few that, despite some temporary interruptions and discontinuation of surgical abortion in two of them, continued to offer abortion services in the years after the revolution. I attended consultations about contraception and abortion, and followed the abortion itineraries of married and unmarried women. I also took part in approximately 15 workshops and seminars organized by three local NGOs active in the domain of women’s health and rights. Although I interviewed numerous doctors, nurses, midwives and activists, the bulk of my ethnographic material originates from daily immersion in the field and the informal conversations I attended or took part in at the clinics, the public hospital and private houses. I did not interview women seeking contraception and abortion care systematically, but I attended their conversations in waiting rooms and during consultations. Conversations were in Tunisian Arabic with clinic users and in French and Arabic with health professionals. I obtained permission to conduct research in the clinics and the public hospital from the National Office of the Family and Population (ONFP), the government agency in charge of sexual and reproductive health services, and the Ministry of Health, respectively. An official letter was circulated in government facilities allowing me to attend consultations and conduct interviews with staff and patients. I negotiated my presence during the consultations with each provider, but I was unable to ask each patient for permission to attend as only a few practitioners introduced me. In most facilities, I was asked to wear a uniform and clinic users thought I was a member of staff. Most women did not seem to pay much attention to my presence, because there were often several health practitioners in the room during the consultations and sometimes even other patients. To introduce myself in a crowded government clinic where time for each patient is very limited would have become unethical as I would have interfered with the personnel’s work and reduced their exchanges with each woman even further. Over 10 months of fieldwork, only one woman asked me to leave the room during her pelvic examination. As it was impossible to introduce myself to each clinic user, I adopted the ethical model of ‘ethnographic consent’ which is a ‘relational and sequential process rather than a contractual agreement’, implying a continuous renegotiation of the relationships between the researcher and her interlocutors (Fassin, 2008: 127). Didier Fassin, who has worked in hospital settings where it was impossible to ask each patient’s permission to conduct ethnographic observation without negatively affecting health professionals’ work and the quality of care, emphasizes that, instead of mechanically applying ethical norms based on abstract principles – usually modelled after biomedical ethics – when conducting ethnographic work, it is more suitable to examine ethical issues ‘each time taken in singular configurations’ (Fassin, 2008: 121). He sees ethics as a practice inspired by the principle of the researcher’s responsibility towards her interlocutors, a difficult and ‘uncomfortable’ task that goes ‘beyond the rule’ (Fassin, 2008: 120). Fassin shows that formal consent can be used as a sort of administrative formality that protects the researchers rather than granting rights to their interlocutors. When I was in Tunisia, I witnessed the formal nature of administration of informed consent in the clinics I attended every day. The most common situation I witnessed was when Tunisian women getting medication abortion had to sign an informed consent form before starting the procedure. Providers handed the consent form to clinic users without any explanation, saying only that they had to sign it to get abortion care. As most women attending public clinics are either very poorly educated or illiterate, it was impossible for them to read the information entailed in the consent form. The staff had no time to explain the information entailed in the consent form to clinic users, and therefore women received no information, although they always signed the consent form.

Fassin developed the conviction that ethnographic ethics cannot be reduced to officially approved protocols after his fieldwork experience in South African hospitals where paradoxes related to the effects of procedures imposed by local ethics committees triggered a collective reflection among the researchers of his team. He realized that the use of oral and written consent among patients and health practitioners had unethical effects on the other anxieties that hindered the possibility of conducting the research. The first effect was related to the fact that, when patients came to the hospital, they were often in a state of moral and physical suffering. Presenting the research and obtaining patients’ consent to participate would have been ethically problematic. In addition, staff were usually under pressure and time was very limited. Gaining patients’ consent would have had immediate consequences on the care they were offered. Finally, presenting the research to patients would often cause a breach of trust between the researcher and the health providers who had become used to her after a long period of fieldwork, reminding them of her presence and interfering with the staff’s work.

The second effect of using (especially) written consent forms was related to the fears and anxieties that specific formulations used in them would create in the anthropologist’s interlocutors. When a relationship of trust had been established, the consent form made the researcher’s interlocutors – especially those at the bottom of the hospital hierarchy who felt in a weaker position – think that they were engaging in a contract rather than signing a document protecting their rights. This generated several refusals to be interviewed whereas the same persons had had informal discussions with the researchers over many months.

Overall, Fassin convincingly shows that ethical protocols presuppose an hypotheticodeductive approach which is antithetical with ethnography, a methodology based on induction and informality, which means that ‘“research” and “daily life” are inextricable’ (Lederman, 2006: 477). Ethics in ethnography should thus be a continuously negotiated practice inspired by the principles of responsibility, respect and beneficence, and adapted to the social and political context in which they are applied. The researcher’s situated position also plays a major role in determining her sense of responsibility and ethical choices as the different stances of the South African and French researchers in Fassin’s team demonstrate. South African researchers refused to sign a collective publication because they deemed it too critical towards the healthcare practitioners with whom they collaborated, whereas French researchers thought it was crucial to show the dysfunctions of the local health system in order to improve care practices (in the patients’ interest) and to respect scientific rigor.

In the Tunisian clinics where I conducted fieldwork, I found myself in situations very similar to those described by Fassin. After having obtained the authorization of local authorities and the consent of health providers, if I wanted to document the situation of health and reproductive health clinics after the revolution of 2011, I had to accept that I could not ask each clinic user whether she was willing to take part in my research as I could often not disclose my identity. It is also important to emphasize that asking clinic users and staff to sign a consent form in a society that, until 2011, was characterized by strict police control and violent social repression could have been interpreted as a dangerous act. Ethical protocols used by many institutions of the Global North are ethnocentric, and presuppose that researchers’ interlocutors are members of democratic societies where they enjoy civic rights. This was, however, not the case in Tunisia at the time of my research and in many other countries where anthropologists work. Although researchers (foreign or local) strive to respect ethical principles that are considered as legitimate, local contexts do not always allow this.

Therefore, even if I was unable to respect all the procedures deemed legitimate in most academic institutions of the Global North, I do not think that I violated the ethics of ethnographic research as Fassin and the position paper of the Swiss Society of Ethnology (of which I am a member) convincingly argue (Société Suisse d’Ethnologie, 2011). I believe that my research, like that of Fassin, invites social scientists to consider ‘the historical and social inscription of the ethical sense’ (Fassin, 2008: 131). It also shows that the North American model of ethics for the social sciences – which is embedded in a specific history (Lederman, 2006) – is not universal and that specific historical and political contexts should be taken into consideration when it comes to its application on the ground.

## The politics of ignorance about medication abortion

The fact that ignorance is a social product requiring several actions (or lack thereof) has become common knowledge in social sciences (Proctor and Schiebinger, 2008, Heimer, 2012), as well as its strong link with the political, social and economic spheres (Tuana, 2004, Tuana, 2006). When it comes to women’s reproduction and sexuality, the specific power relations generated by the local gender system must also be considered in societies dominated by patriarchal logics that shape relationships between individuals according to their sex, class and race. Reproduction and sexuality determine rigid forms of surveillance and control over women’s bodies and conduct in Tunisia. Despite a Code of Personal Status (CPS) that, for many years, was one of the more liberal in the Arab world (Chekir, 1996, Bouraoui, 2001, Charrad, 2008), women are still discriminated against by law and subject to various forms of violence (Ben Achour, 2016). The postcolonial state decriminalized abortion in 1973 for all categories of women without marital consent in the framework of demographic policies aimed at reducing the high fertility rate, which was deemed detrimental to the modernization and economic development of the country (Gastineau and Sandron, 2000; Lococh and Vallin, 2001). Until the end of the century, surgical abortion was the only authorized technology, as stated by Article 214 of the Penal Code. In the mid-1990s, the first clinical trials on medication abortion were conducted in Tunisia under the umbrella of the World Health Organization (WHO) and later the Population Council and the New-York-based NGO Gynuity Health Project (Blum et al., 2004, Hajri et al., 2004). By this time, a large number of clinicians, some jurists and many politicians opposed the introduction of this technology in the country. Doctors were afraid that it would deprive them of control over this act, as paramedical personnel can easily supervise it. Some jurists insisted that the law does not allow the use of medications to perform abortions or the possibility of women doing so at home (two features of medication abortion) because the Penal Code states that abortion cannot be performed by administering ‘beverages, medicines or any other means’ (Penal Code, Article 214) and shall take place in a licensed medical facility. Politicians were preoccupied by the possible freedom that women could enjoy by using a less invasive and less medically controlled technology. These actors were all in positions in which they were able to exert some form of power over women’s sexual and reproductive behaviours. The opposition to the introduction of medication abortion in Tunisia was inscribed in a ‘configuration of interests’ (Tuana, 2006: 4) that did not allow the mentioned groups of actors to acknowledge that it is a safe and standardized method promoted by WHO (World Health Organization, 2014), less expensive than surgical abortion, requires limited medical supervision and is favourably accepted by women (Blum et al., 2004, Hajri et al., 2004).

For all these reasons, the introduction of medication abortion should not have elicited such opposition in a country with scarce resources where abortion had been legal for several decades and was offered free in government facilities to all women. Instead, the National Association of Obstetricians and Gynaecologists opposed its introduction, allegedly because its representatives considered it dangerous (Hajri et al., 2004), evoking the possible catastrophic haemorrhage caused by medication abortion that could kill or endanger the health of Tunisian women (interview with Selma Hajri, the clinician who introduced medication abortion in Tunisia, 2013). Most doctors chose to ignore the existence of clinical studies proving its efficacy and safety. Jurists used the formal aspect of the Penal Code to oppose medication abortion instead of trying to adapt the text of the law to the available new technology, which includes the administration of two medicines (mifepristone and misoprosrol) at an interval of 48 h to induce the expulsion of the uterine material. Most women included in the clinical trials on medication abortion expressed their preference for a home procedure (Blum et al., 2004), which international experts consider safe. Despite widespread opposition, thanks to the support of the then-President of ONFP, Nabiha Gueddana, and a few obstetricians working in large government hospitals (Hajri, 2004), local authorities approved medication abortion in 2001. Although the law was not changed, a circular letter allowed government and private facilities to adopt medication abortion. ONFP organized training courses designed for medical and paramedical personnel in the public and private sectors (Hajri, 2004, Hajri et al., 2004). However, many providers remained sceptical or opposed to medication abortion even after its introduction in the country, especially for unmarried women (Blum et al., 2004). Participation in training courses notwithstanding, clinicians in the private sector were not keen to adopt the new technology because it made their intervention minimal, limiting the economic benefits of the procedure. Surgical abortions continued to be the most practised method in the private sector, not only for financial reasons but also for organizational reasons. As the medications used for abortion have to be provided by the Central Pharmacy, a government institution, private clinics should make them available to private physicians using their technical facilities. This goes against the interests of private clinics and doctors for whom surgical abortions are much more lucrative and do not require a follow-up after 2 weeks. Individual private doctors cannot obtain the medications for abortion, and clinics are not interested in providing them to private doctors. The organization of the private sector makes ‘strategic ignorance’ of medication abortion conducive to its good functioning (McGoey, 2012, Tuana, 2006). Even if many private gynaecologists received training on medication abortion in the early 2000 s, in the years after the revolution, they seem to have forgotten the correct protocol or even that they had the possibility to purchase the medications to perform it, as many health providers I met during the NGO seminars I attended told me. The Tunisian situation is one in which ‘what was once common knowledge’ has been ‘transferred to the realm of ignorance (…) because such knowledge is no longer seen as valuable, important or functional’ (Tuana, 2004: 195). A gynaecologist with whom I collaborated in several workshops for an NGO and who became a friend told me that many of his colleagues had intentionally ignored or neglected medication abortion because they felt deprived of their medical authority, whereas younger practitioners never received training on this technology because ONFP had stopped offering it. This is another important issue that helps explain why knowledge still owned by some has not been distributed among many others (Heimer, 2012). On one hand, ONFP ‘retained’ knowledge on medication abortion; on the other hand, a ‘process of unlearning’ took place among providers (Tuana, 2006: 2) over the last 15 years. One of the reasons why ONFP stopped training providers in medication abortion is that once the demographic transition took place in Tunisia in the late 1990s, family planning policies lost their importance, and much less attention and resources were devoted to them. Family planning policies (including abortion) had been a political priority for the independent state from the mid-1960s to the mid 1990s (Vallin and Lococh, 2001). Tunisia created ONFP and a network of family planning clinics in all areas of the country, and launched mass campaigns to encourage the use of contraception, instituted financial incentives for health providers and governorates lowering their fertility rates, etc. (Foster, 2001). The realization of the demographic transition also explains why most public hospitals and several ONFP clinics stopped offering abortion care over the course of the 2000s despite the legal obligation to do so (Association tunisienne des femmes démocrates, 2013). Ideological, religious, economic and organizational reasons were also important in the decrease of abortion services in Tunisia. The spread of religious and social conservatism in the 2000s, severe understaffing, and lack of equipment and medicines in the public health sector contribute to explaining the production of ignorance about pharmaceutical abortion (Foster, 2016, Maffi, 2020).

I will now turn to the varieties of ignorance existing in Tunisia about medication abortion, and investigate them according to the fields in which they are produced. I will consider several actors who participate in or resist this production: women/patients, health practitioners, social workers or administrative personnel, government institutions and NGOs.

## Ignorance of the law

An important field of ignorance concerns the law. There are many forms of ignorance among the mentioned categories of actors, some of which are wilful and others are the result of negligence, misinterpretation or sequestration of knowledge. Most women who attend public facilities have a low level of education and belong to the poor strata of the population. They usually do not understand that abortion is their right, and that providers should offer them this service and see it more as a ‘concession’ (Amuchastegui and Flores, 2013: 921) or a ‘moral debt’ to the provider (Krauss, 2018: 694). After the revolution, many women were turned away in the public sector (Hajri et al., 2015, Maffi and Affes, 2017) and had trouble obtaining appropriate and timely abortion care because health providers or administrative personnel refused their requests. A considerable number of women I met had been to two or three facilities before getting abortion care, causing them anxiety, fear, loss of time and considerable costs in relation to their meagre income. Ignorance of the law among women and health providers was partly the result of a lack of information as, according to the numerous testimonies I collected among both groups of actors over the last 15 years, the state has not taken any steps to disseminate the necessary information, partly as the result of staff negligence in health facilities. Few practitioners were aware of the content of the ONFP Reference Manual, which is updated regularly and which contains medical as well as legal information about the services available in its clinics. During the seminars organized by one of the Tunisian NGOs with which I collaborated, it was common for professionals working in government facilities to ask explicitly for information about the abortion law and even details about the protocols of medication abortion.

I witnessed a case of ignorance of the law by staff in the large public hospital where I conducted fieldwork. The social workers who investigated the situations of unmarried patients coming for abortions did not apply the law promulgated in 2004 defining women’s age of majority. Before this law, women were considered adults at the age of 20 years, whereas after its announcement, they were considered to have attained majority at 18 years. In 2013–2014, hospital social workers treated women aged between 18 and 20 years like minors, asking them to obtain the permission of a guardian to access abortion care. This request often generated dramatic situations, as the young women were usually unmarried, and their sexual relationships were socially and religiously condemned (Maffi and Affes, 2019). Social workers deliberately ignored the 2004 law, insisting that it did not entail the notion of sexual majority, which the CPS establishes at 21 years for unmarried women. Two or three practitioners out of the dozens I met, in speaking to women who were turned away by other health professionals, insisted that they should return to those facilities and claim their right to abortion or complain to local authorities about their situation. Many poor and uneducated women protested that they only wanted to get abortion care and could not do what the midwives suggested, or said that they only prayed for the provider to accept their request for an abortion. For most of these women, the priority was to get abortion care rather than claiming their rights, despite the importance that the discourse of justice, dignity and rights had gained with the 2011 revolution. Clinic users were well aware that the hostility of many health providers to abortion could not be overcome by claiming their rights, and that they were caught up in an unequal power relationship.

Many practitioners I worked with refused to offer abortion care to women coming for repeated abortions – usually called ‘recidivists’ – especially if they refused to adopt a reliable biocontraceptive method (Lock and Nguyen, 2010). Providers wrote in their medical files that they could not have any further abortions as they had already had several. It was a widespread practice specifically related to the technology of medication abortions, as the procedure does not require women to return to the clinic after the administration of treatment, meaning they avoid the personnel’s control. This is very different from surgical abortion; in the past decades, providers were able to insert intrauterine devices (IUDs) in all women during abortion procedures, even without their consent (Association tunisiennes des femmes démocrates, 2013). As in other national contexts, such as Mexico (Krauss, 2018) and France (Memmi, 2003), paternalistic attitudes towards patients who requested abortions were repeatedly justified by the idea that these women were undisciplined and irresponsible (Maffi, 2020). In this case, the law was deliberately ignored in the name of the good of the patient and of the nation, as some providers insisted that all these abortions were a waste of money and time. In two of the facilities where I did fieldwork, the doctor in charge of the clinic did not know about these practices or pretended not to know to avoid conflict with the staff or being the target of symbolic or even physical violence. After the revolution of 2011, some ONFP clinics were attacked and devastated, and groups of Islamic extremists had threatened the personnel; as such, some clinicians were afraid this could happen again (interview with two health clinicians in charge of two government facilities, 2013). In another facility, a practitioner responsible for abortion care had received a threatening letter and repeated violent verbal exchanges with some of her colleagues who condemned her professional activity.

One of the NGOs with which I collaborated as a trainer for several months organized regular seminars for personnel working in government facilities offering abortion care. It provided participants with information about Tunisian abortion law and women’s sexual and reproductive rights, spreading concepts and knowledge with which local staff were sometimes unfamiliar. In 2013–2014, these seminars were organized regularly in various regions of the country in collaboration with ONFP and the Ministry of Health to spread correct information among the staff of government facilities about their professional duties and to clarify their ethical values. Participants – recruited on a voluntary basis – attended trainers’ presentations on various topics (mainly on abortion), took part in common discussions, and exchanged on their personal and professional trajectory (see below).

Despite their commitment to reaffirm women’s sexual and reproductive rights, the NGO’s trainers stressed that, as the postrevolutionary ideological environment made people hostile to this discourse, they should avoid referencing that concept and rather insist on the health argument to justify abortion. By encouraging legal knowledge among health providers, the NGO was, in a way, perpetuating ignorance about it to make their message socially acceptable. This example shows that the border between knowledge and ignorance is continually negotiated (Gross and McGoey, 2015), and that ignorance is produced ‘to avoid rupturing with certain social scripts’, even by actors who aim to spread knowledge in a domain of ignorance (Tuana, 2004: 224).

## Ignorance of medical procedures

Another domain of ignorance specifically concerns health professionals and what they know (or do not know) about the protocols of medication abortion. First, neither doctors nor midwives learn about medication abortion during their training, as mentioned above. All midwives I met – in ONFP clinics, in the hospital and at NGO training seminars – declared that they had learned this technology after starting work in facilities that offered it. Some health professionals, to justify their hostility towards (medication) abortion, stated that they were trained to attend births rather than to kill babies (in Tunisian Arabic, ‘saghir’ or ‘bébé’). As for the gynaecologists, in the large university hospital where I carried out my participant observation, several resident doctors came to the family planning unit to receive training in contraception and abortion, but stayed for only 1 or 2 h and then left, asking the midwife to put a stamp in their college transcripts proving that they had completed the training. The lack of concern about abortion and contraception in gynaecological training is common in Tunisia and in most other countries. The emphasis is on pathology, surgery and ultrasound imaging rather than abortion and contraception, as the latter are seen as unimportant and usually relegated to midwives in the public sector (interview with an experienced gynaecologist, previous head of one of the units of the larger maternity hospital in Tunis and professor at the Medical School, 2013). Similarly, a professor at the Department of High School for Health Science and Techniques of Tunis – with whom I collaborated regularly – who is in charge of midwives’ training confirmed that medication abortion is not part of the curriculum (interview, 2014).

The exclusion of medication abortion from professional training has an important impact on providers’ consideration of it as a minor or even despicable topic (Kumar et al., 2009). Additionally, training on pharmaceutical abortion for medical and paramedical staff in public and private facilities was discontinued a few years after the introduction of this technology in Tunisia (interview with Selma Hajri, 2013). The ‘distributed ignorance’ among providers meant that many private doctors, and sometimes even ONFP personnel, did not know the correct protocol for medication abortion. Several women I met in ONFP clinics told me about failed medication abortions in the private sector, where they had received various medicines that did not cause expulsion of the pregnancy.

According to some clinicians in charge of medication abortion, even upon its introduction, many ONFP providers did not easily accept training related to medication abortion and, after the revolution of 2011, several ONFP officers had to negotiate with their clinics’ personnel to offer abortion services because the latter openly opposed them (interview with Nabiha Gueddana, former President of ONFP, 2013). Ignorance was also fostered by providers who transferred women seeking abortion care to tertiary care hospitals, allegedly because of counterindications for pharmaceutical abortion such as allergic asthma, obesity, light anaemia or previous caesarean uterine scars. Some ONFP personnel I observed also told patients that repeated medication abortions may cause sterility, severe haemorrhage or uterine cancer. These arguments were used to discourage and even frighten ‘recidivist’ patients who, instead of using reliable contraception, had come repeatedly for abortion care. Whether those providers believed what they said was often unclear to me, but their words showed that they did not know or voluntarily neglected medical evidence and WHO’s documentation about medication abortion. Unmarried women were the main targets of these discourses because clinicians considered them more irresponsible due to their (often) young age or because they had sexual intercourse outside marriage. Unmarried patients were also more afraid than married patients about becoming sterile, as procreation is a central feature defining women’s identities.

## Ignorance about contraception

Ignorance about contraception among health practitioners and women also contributes to producing ignorance in the domain of reproductive health, thus increasing abortion rates, as many women do not know how to use contraceptive methods. Except for a few recent attempts, men have never been involved in contraception in postcolonial Tunisia, and reproductive and sexual health clinics are almost exclusively feminine spaces. Ignorance among providers concerned the use of IUDs and, for some, birth control pills. As the IUD is the most common contraceptive technology in Tunisia (Dimassi et al., 2017), health providers’ ideas and use of it were the cause of many unwanted pregnancies and, therefore, of abortions. IUDs were never offered to unmarried women and only rarely to women who had one child; many practitioners I met believed that the ideal number of children in a family is two or three. They seem to have inherited the distrust of the IUD that is historically related to the scandal of the Dalkon Shield, a specific model of IUD that killed many American women or made them sterile in the 1970s and 1980s (Hartmann, 1995, Takeshita, 2010). Practitioners also had various ideas about how long women had to wait for an IUD after a caesarean section. Some explained to patients that they should wait 6 months, others said 3 months; the Sexual and Reproductive Health Reference Manual of ONFP (2014) recommends an interval of 40 days. Therefore, many women who did not want to use hormonal methods had to wait several months and often became pregnant as they thought that, because they were breastfeeding, they could not conceive. Several providers I met were also convinced that the pill should not be administered to women before marriage (and the birth of at least one child), as they could become sterile. This shows again that the production of ignorance in the domain of contraception is related to the lack of training and sequestration of knowledge considered unimportant. The fact that biomedical contraception concerns women’s bodies exclusively confirms the gendered nature of biogynaecology in Tunisia and elsewhere (Van Kammen and Oudshoorn, 2002), and that the production of ignorance is the result of a configuration of male-dominated interests (Tuana, 2006).

Women attending public facilities were often ignorant about biocontraception and did not know how to use the pill. Some thought that they should take it only if they had sexual intercourse; others thought that if they had sex only once or twice a month, they could not get pregnant; and others simply forgot to take the pill every day or took it at different times of the day. This was the case with a very low-dose pill that was freely available in the public sector, which needed to be taken at the same time each day to avoid pregnancy. Several women had to give up using IUDs because of opposition from their husbands, who complained about feeling the string attached to the device during intercourse, and were unable to replace it with another acceptable method. Most methods that were available free of charge in the public sector had side effects that many women did not easily tolerate. Therefore, a significant number of women used the calendar method, which did not always prevent unwanted pregnancies. Women ignored the correct use of biocontraception because they were often uneducated, had inaccurate knowledge of female physiology (Maffi, 2020), and did not receive clear explanations at the clinic about how the various methods work (Ben Dridi and Maffi, 2018). Women’s ignorance was thus a form of distributed ignorance due to organizational (lack of time to give explanations), sociocultural (lack of formal education) and biomedical (characteristics of contraceptive methods) reasons.

## Ignorance about women’s attitudes and motivations

Another form of ignorance among providers concerned women’s motivations to seek abortion and their attitudes towards early pregnancy. Work conditions as well as the moral or religious stances of providers produced this particular form of ignorance. Many practitioners I met were convinced that women considered medication abortion to be an easy solution, often preferable to contraception because it is less invasive than surgical abortion. Clinicians often told me that medication abortion had brought about the banalization of this act – that many women do not think they are ‘killing a baby’ if they just have to take some tablets – and that it has caused a tremendous increase in the abortion rate. All these allegations are unfounded if we consider that the number of abortions has remained stable in the public sector since the introduction of medication abortion, that most women I met did use contraception – according to the World Bank, the contraceptive prevalence in Tunisia was 62.5% in 2012 (World Bank, 2012) – and that the procedure they had to go through to get an abortion was troublesome and thus not easily undertaken (Hajri et al., 2015, Maffi and Affes, 2017, Maffi and Affes, 2019). In addition, many providers, especially after the revolution, were openly hostile to abortion for moral or religious reasons. Secular providers trained in the biomedical visual culture were used to perceiving early embryos as babies (Maffi, 2020), and religious practitioners presented abortion as an illicit (‘haram’) act, sometimes reading passages of the Quran in front of patients. High pressure in public facilities made it very difficult for providers to take the time to listen to women’s motivations and feelings when they came for abortions. Many women felt conflicted about ending their pregnancies and wanted to do it as soon as possible. They thought that until 40 days had passed (some thought up to 3 months), what they were carrying was not yet a human being, as ensoulment (divine insufflation of the soul into the products of conception) had not yet happened (see below). Tunisian law does not provide for a compulsory interview with a woman who wants to abort, so no institutional space exists where providers and women can talk about abortion. The above-mentioned NGO organized training seminars where health providers were invited to discuss their ideas and values related to abortion. They were able to express their opinions freely and talk about their personal and professional experiences with abortion. In these spaces, they also received legal, medical and religious information about abortion in Tunisia, other Arab countries and the rest of the world. It was often the locus where sequestered knowledge about (medication) abortion was distributed, replacing participants’ ignorance: for instance, statistical and legal knowledge about abortion in Tunisia, Africa and the Middle East; abortion laws in Islamic states; data on maternal mortality related to abortion across the world; and true stories about the deaths of women resorting to unsafe abortion. Role-playing games were also organized to push providers to identify with patients seeking abortion care in which they had to play the role of the woman, the practitioner refusing to offer abortion care or the practitioner trying to help the patient. Training seminars also offered extensive information about the status of abortion in Islamic legal and religious texts of the Sunni tradition. This was deemed a crucial topic because of the spread of very rigorist Islamic opinions nurtured by preachers and television broadcasts of satellite channels in the Gulf States. According to the testimonies of the dozens of participants I met, the NGOs’ seminars were innovative spaces where they were able to freely discuss their personal and professional experiences of abortion, their values about the beginning and ending of life, the rights of women in the domain of sexuality and reproduction, and their religious beliefs, but also to acquire legal, medical and Islamic knowledge based on reliable sources. For some practitioners who declared that they were against abortion on the first day of the seminar, the information received and the discussions with the trainers and other health providers were very important and contributed to modify their behaviour, as I observed in two facilities where I conducted fieldwork. These providers stopped turning away women seeking abortion care and changed their discourse, saying that, although they personally deemed abortion a despicable act, they understood that it could help poor and/or unmarried women to save or improve their lives.

## Ignorance about the plurality of Islamic discursive traditions

As a result of weak control over staff working in government health facilities, after the revolution, several practitioners began to behave according to their personal beliefs rather than respecting the law that grants all women access to abortion and contraception. The personnel of many clinics began to mobilize religious arguments to refuse abortion care drawing on the conservative view circulating during this period. Health practitioners and women seeking abortion care often ignored the positions of the four major legal schools of Sunni Islam (Shafiite, Malikite, Hanafite and Hanbalite) on abortion. The personnel of many clinics – including the receptionists and health educators who had the power to turn women away before they had even seen a health provider – were convinced that abortion is ‘haram’ (religiously illicit). Several used to tell women seeking abortion care that it was forbidden by religion as a sin and the murder of a human being. They adopted and spread a particular doctrinal opinion, neglecting a much more complicated tradition in which there are various stances within and between the mentioned legal schools. Some ‘ulama’ (religious scholars) consider abortion religiously acceptable up to 40 days, others up to 80 days and still others up to 120 days after conception. Referring to Surah 23 of the Quran and a well-known ‘hadith’ (saying or action of Prophet Muhammad), they attribute various durations to each phase of embryonic development (Musallam and F. , 1983, Bowen, 2002; Shapiro, 2013). Overall, the moment the product of conception becomes a human being coincides with ‘the inbreathing of the spirit’ (Katz, 2003: 30) when the soul enters the fetus’ body. For several Islamic scholars, before ensoulment, the fetus is not a human being, and thus abortion is not forbidden nor a sin. However, for some legal schools (Malikite and Shafiite), the products of conception are a human being immediately, and thus they prohibit abortion, although the Quran makes no mention of this act. The women I met carried various interpretations of abortion. For some, a pregnancy is a ‘baby’ at the beginning; for others, it becomes a ‘baby’ after 4 weeks; and for some, it was not a ‘child’ until the end of the first trimester. Hence, they did not all speak and feel the same way about abortion, although generally, the women I observed were not particularly upset when they took the abortifacients. In the clinics where I conducted my research, married women had to wait together in a room for some hours before going back home. They often talked to each other in a relaxed and even cheerful way. After the revolution, a plurality of religious opinions co-existed with the Tunisian abortion law that allows abortion until the end of the first trimester, and even further if the mental and physical health of the mother is endangered. It is worth mentioning that when the law was promulgated in 1973, it was legitimized through references to legal religious opinions allowing abortion until 120 days after conception as opposed to referring to women’s rights to control their bodies. While uncertainty about the law and rigorist religious opinions about abortion were disseminated after 2011 (Maffi, 2020), in the seminars for health providers organized by the mentioned NGO, trainers explained that there are various interpretations of abortion in Sunni Islam and that religion is a private matter that should not be mobilized in healthcare facilities. They were trying to resist ignorance about Islamic stances towards abortion, offering a more nuanced and rich view of the religious tradition. At the same time, they intended to reinforce the secular attitude of providers because, as mentioned above, after 2011, some of them had started to use religious arguments to turn women away or persuade them that they were committing a sin when they sought abortion care. In the first years after the revolution, reading the Quran in front of patients seeking abortion care became a widespread practice aimed at persuading them that they were committing a sin, as many of my interlocutors told me. The electoral victory of the Islamist party Ennahdha in October 2011; the legitimization and circulation of very conservative religious ideologies from Saudi Arabia, Qatar and Egypt (silenced by the previous regime); and the political instability and social transformations following the revolution all contributed to producing new forms of ignorance about Islamic positions towards (medication) abortion in a country where this act was decriminalized more than 40 years ago with the support of local religious scholars. The Islamic discursive tradition, which the modernist elite who ruled independent Tunisia had mobilized to justify progressive social and political reforms, became a source of legitimation for conservative discourses and behaviours infringing the state laws after the revolution of 2011. Turning away women seeking abortion care claiming the re-establishment of polygamy and the complementarity between men and women were but some expressions of the conservative use of a plural Islamic discursive tradition.

## Conclusion

In this article, I have explored the various forms of ignorance produced in Tunisia about medication abortion since its introduction in the early 2000s. These forms of ignorance concern the law, biomedical technologies (contraception and abortion), health providers’ perceptions of women’s attitudes towards abortion, and Islamic positions on this technology among clinic users and staff. Upon its introduction, medication abortion was met by strong opposition from several groups of (mainly male) actors: gynaecologists, jurists and politicians. Their explicit arguments against medication abortion were dissimilar, but they all agreed implicitly on the fact that it went against local social values and norms that imply the submission of women to a rigid regulation of their sexual and reproductive conduct. Their opposition was built on the ignorance of the medical, economic and social benefits of pharmaceutical abortion; ignorance that was – to some extent – wilfully cultivated. Organizational and systemic factors also played roles in the production of ignorance about medication abortion. Health providers’ lack of training about contraception and abortion, the private sector’s interests in offering surgical abortion alone, the difficulties of getting the medications necessary to perform medication abortion, and the spreading of incorrect information about counterindications and health effects of the technology were forms of sequestered knowledge, distributed ignorance and voluntary deception. Additionally, the disappearance of states’ demographic concerns in the late 1990s and the lack of resources in the public health sector contributed to the neglect of (medication) abortion and the discontinuation of this service in most government hospitals. Finally, the emergence and legitimation of very conservative religious ideologies about women’s status, rights and sexual conduct after the revolution contributed to the production of ignorance about medication abortion. One of the NGOs with which I collaborated organized various activities to resist the transfer of knowledge about (medication) abortion to the realm of ignorance, and to circulate knowledge about the legal, medical and moral aspects of this technology among healthcare providers as well as a larger audience. Although stigmatization and moral condemnation of abortion were already present before 2011, the success of very conservative Islamic views made health providers more hostile to this act and turned many women’s abortion itineraries into an obstacle course. Even if the Tunisian NGO with which I collaborated wanted to fight against this phenomenon, it had to make some compromises and silence the discourse on women’s sexual and reproductive rights to make its campaign in support of abortion acceptable for a large number of conservative health providers.

## Declaration

The author reports no financial or commercial conflicts of interest.
